# Supplementary material for: Resolving the spatial architecture of myeloma and its microenvironment at the single-cell level
Source: Nat Commun. 2023 Aug 17;14:5011. doi: 10.1038/s41467-023-40584-4 (PMC10435504; doi:10.1038/s41467-023-40584-4)
Supplement: Supplementary file 1 — Supplementary Information [file 41467_2023_40584_MOESM1_ESM.pdf]

Resolving the Spatial Architecture of Myeloma and its Microenvironment at the Single-Cell Level

Supplementary information

Supplementary Figures

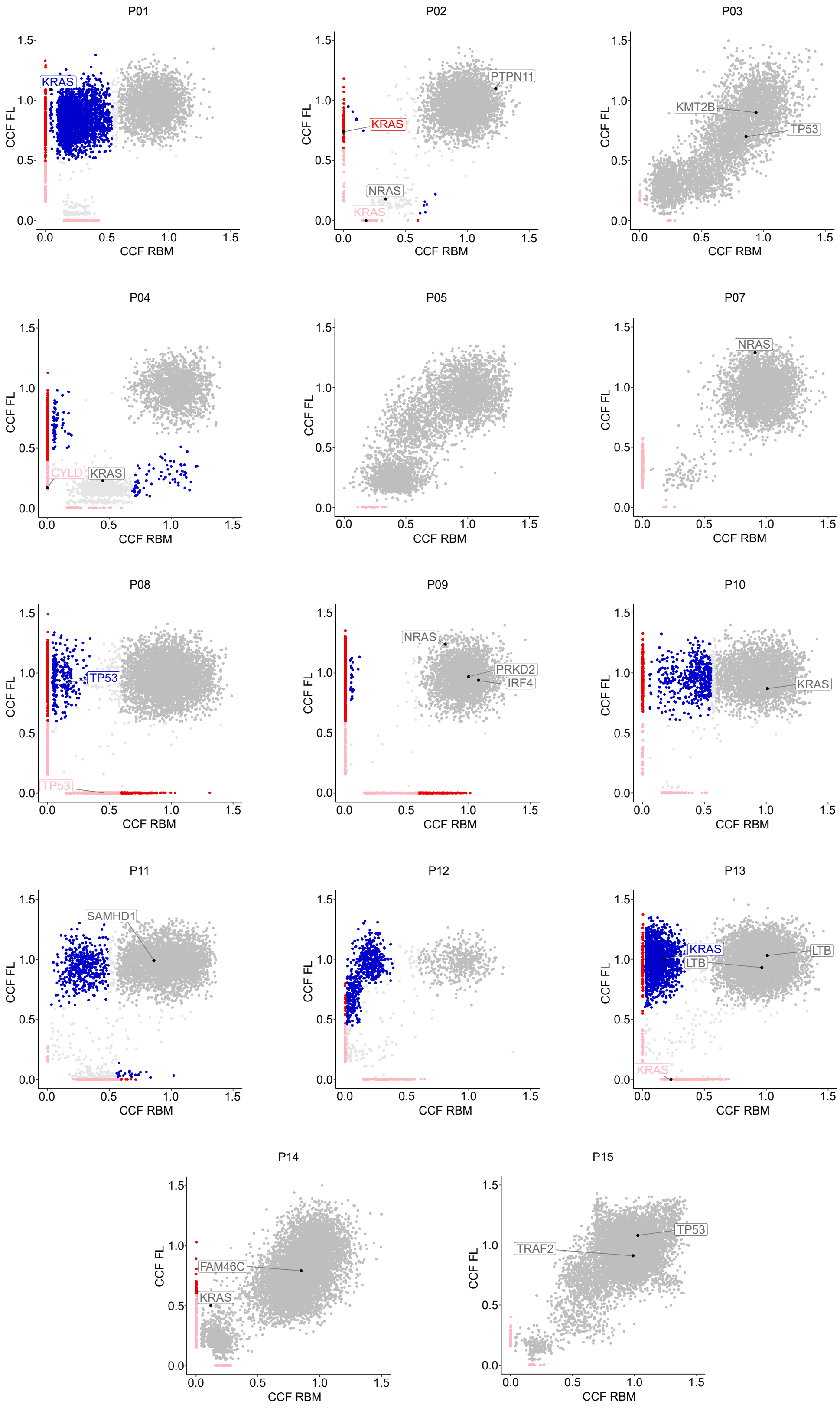

Suppl. Fig. 1: Cancer clonal fraction (CCF) plots of paired samples. Major and minor events between paired random bone marrow (RBM) and focal lesions (FL) were discriminated based on the 95% confidence intervals (95% CI). Mutations were classified as major (*red*), if the upper band of the 95% CI was ≥ 1, and minor (*pink*) otherwise. Furthermore, major mutations with a 3-fold enrichment between the paired samples were classified as enriched (*blue*). Nonsynonymous SNVs in driver genes[1] are highlighted. Source data are provided as a Source Data file.

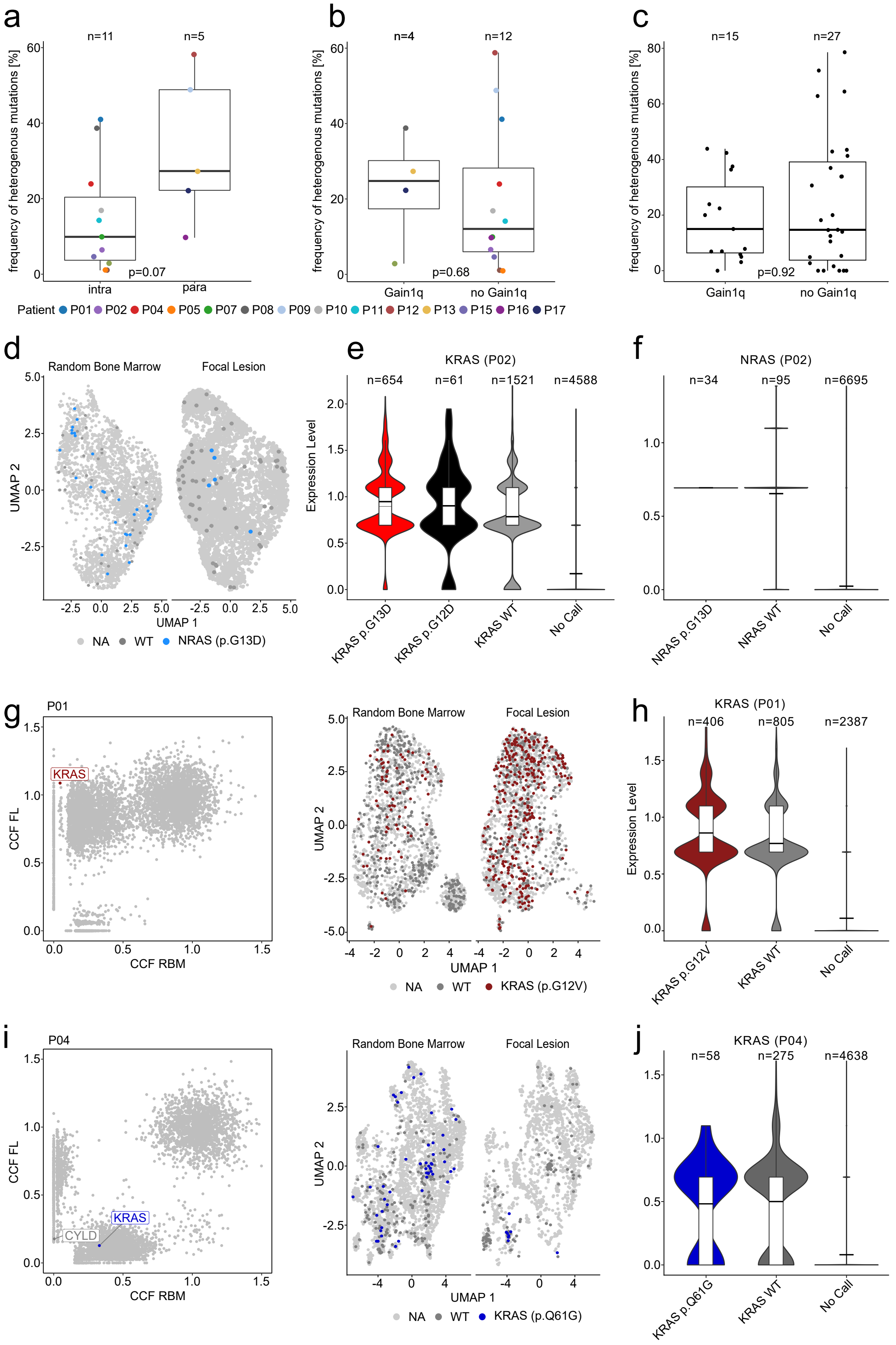

(legend on next page)

Suppl. Fig. 2: Frequency of heterogeneous mutations in patient subgroups and *KRAS* mutation calling in whole genome and single-cell RNA-seq data. (a) Frequency of heterogeneous mutations (unshared+enriched) in intra- vs. paramedullary samples (n=16 patients). The p-value was calculated using two-sided Wilcoxon rank sum test. (b-c) Frequency of heterogeneous mutations (unshared+enriched) in patients with gain 1q vs. patients with no gain 1q in the Heidelberg cohort (b, n=16 patients) and the UAMS patient cohort (c, n=42 patients). The p-value was calculated using two-sided Wilcoxon rank sum test. (d) Uniform Manifold Approximation and Projection (UMAP) and single-cell calls for the NRAS (p.G13D) mutation in patient P02. Blue dots denote cells with a NRAS mutation, dark gray dots denote cells with NRAS wild type (WT), light grey dots indicate cells with no NRAS call. (e-f) Violin plot of KRAS expression in cells with or without called KRAS-mutation (e) and NRAS expression in cells with or without NRAS-calls (f) in patient P02. (g) Whole genome sequencing (WGS) cancer clonal fraction (CCF) plot for single-nucleotide variants (SNVs) in paired random bone marrow (RBM) and focal lesion (FL) specimens from patient P01. The patient presented with *KRAS* (p.G12V), which was a major mutation at the FL site and a minor mutation at the RBM site. The mutation was called using scRNA-seq and positive calls are marked in the UMAP of paired RBM/FL scRNA-seq data (*red*, right panel). (h) Violin plot of expression of KRAS in cells with or without called KRAS-mutations in patient P01. (i) The same plots are shown for patient P04. The patient presented with a KRAS (p.Q61G) mutation, which was more frequent at the RBM site. Positive calls are depicted in darkblue in the scRNA-seq UMAP. (j) Violin plot of expression of KRAS in cells with or without called KRAS-mutation in patient P04. The boxplots show the median and the interquartile range, while the upper and lower whiskers show the highest and lowest value. Source data are provided as a Source Data file.

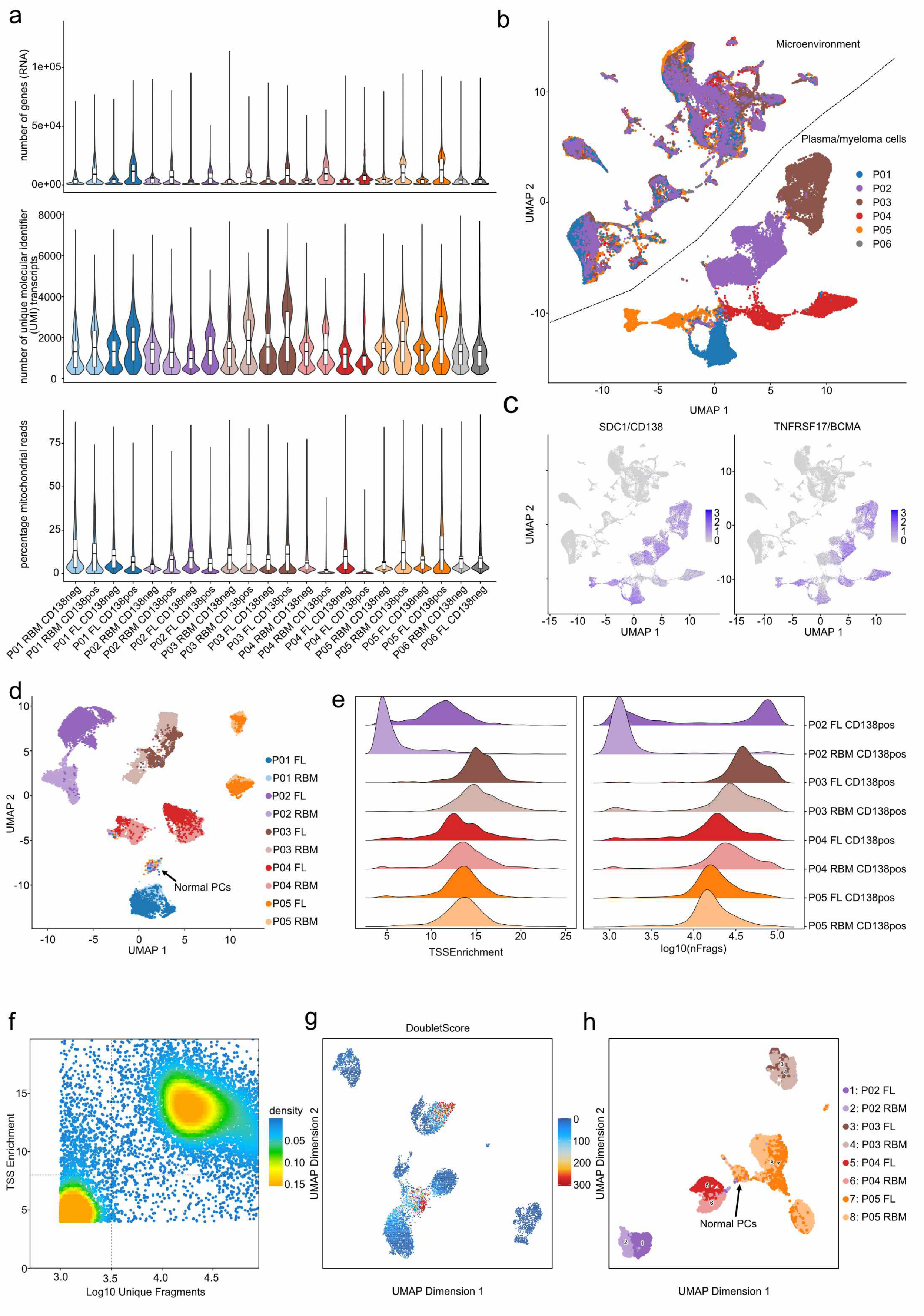

(legend on next page)

Suppl. Fig. 3: Quality control of scRNA-seq (a-d) and scATAC-seq (e-h) data. (a) Violin and box-whisker plot of the number of informative genes, number of unique molecular identifier (UMI) transcripts and percentage of mitochondrial reads per single cell per experimental sample to define quality control (QC) thresholds. Cells with more than 5% mitochondrial RNA, less than 200 or more than 5000 expressed genes were removed. Cell numbers before and after QC are provided in Suppl. Table 7. The boxplots show the median and the interquartile range, while the upper and lower whiskers show the highest and lowest value. (b) UMAP of all 74,631 cells after QC coloured by patients. (c) Feature plot of the expression of the two plasma cell marker genes CD138 (SDC1) and BCMA (TNFRSF17) to split the dataset into plasma cells (CD138-positive) and cells from the tumor microenvironment (TME, CD138-negative). (d) UMAP of CD138-positive myeloma/plasma cells from paired random bone marrow (light colors) and focal lesion (dark colors) specimens from 5 newly diagnosed patients. The multicolored cluster (arrow) corresponds to normal plasma cells which were used as a reference for CNA-calling. (e) Distribution of transcription start site (TSS) enrichment scores and number of unique nuclear fragments for each single cell before filtering (scATAC-seq). (f) scATAC-seq QC filtering plot for all RBM and FL patient samples showing the TSS enrichment score vs unique nuclear fragments per cell. Dot colour represents the density in arbitrary units of points in the plot. Dashed lines represent the filters for high-quality scATAC-seq data (3,000 unique nuclear fragments and TSS score greater than or equal to 8). (g) UMAPs of scATAC-seq data coloured by the per-cell doublet score to define filtering thresholds. (h) UMAPs of the filtered scATAC-seq data coloured by patient and sample. The arrow marks the cluster of normal plasma cells used as reference for CNA-calling.

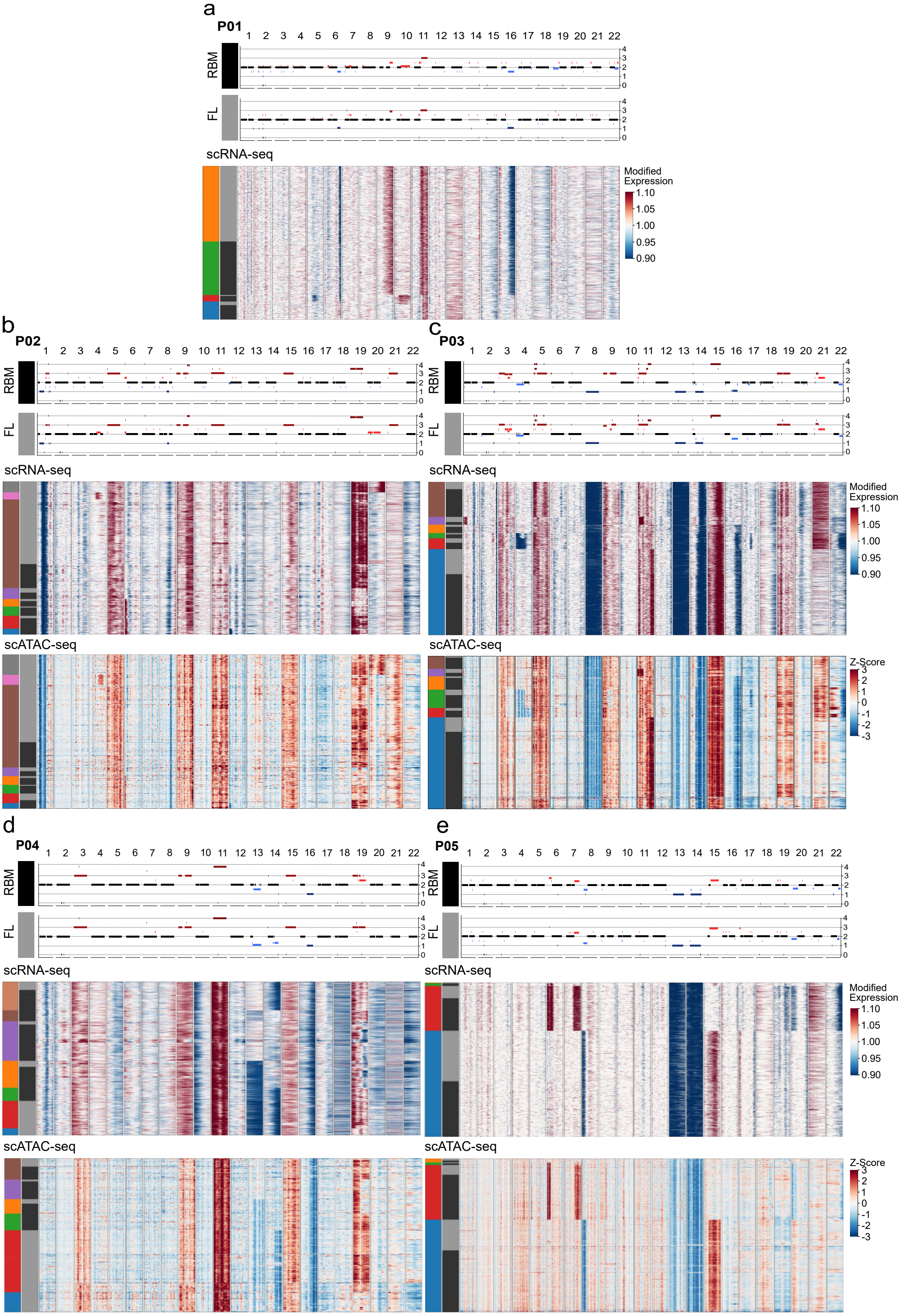

(legend on next page)

Suppl. Fig. 4: Copy number aberration defined subclones in single-cell data. (a)-(e) In the upper panel of each plot the whole genome sequencing chromosomal profiles for each autosomal chromosome in paired RBM (*black* bar) and focal lesion (FL) (*gray* bar) specimens are depicted. *Light red* and *light blue* denote subclonal chromosomal gains and losses, respectively. Clonal events are marked with *dark red* and *dark blue*. To identify subclones, the average relative gene expression/accessibility in regions impacted by subclonal events was used for supervised clustering of scRNA-seq (middle panel) and scATAC-seq (lower panel) data of paired RBM (*black* bar) and FL (*gray* bar) samples. In the heatmaps *red* and *blue* signals correspond to higher and lower gene expression/accessibility, respectively. The detected subclones are depicted on the left side of the two heatmaps in different colors. For P01 no scATAC-seq data was available.

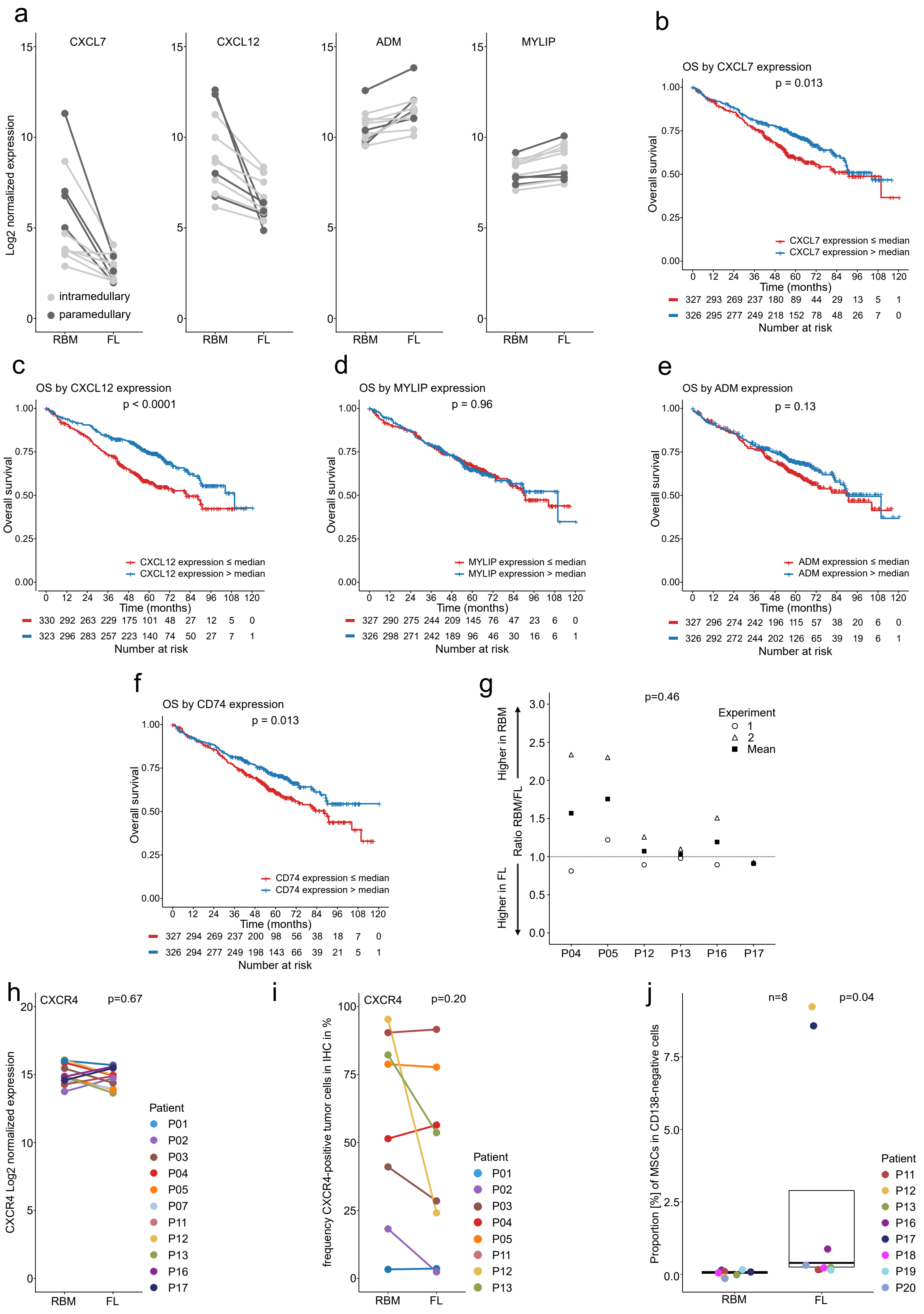

(legend on next page)

Suppl. Fig. 5: Comparison of chemokine expression in different subgroups and expression differences in RNA-seq, ELISA and immunohistochemistry (a) Comparison of gene expression between intra- (light grey) and paramedullary (dark grey) samples for CXCL7 (PPBP), CXCL12, ADM and MYLIP. (b-f) Overall survival (OS) of 653 newly diagnosed multiple myeloma patients[2] stratified by the median expression of (b) *CXCL7* (*PPBP*), (c) *CXCL12*, (d) *MYLIP*, (e) *ADM* or (f) *CD74*. P-values were calculated based on log-rank test. (g) Protein expression of CXCL12 based on ELISA. Ratios between random samples and focal lesions are plotted for 6 patients (n=2 replicates, the p-value was calculated with a linear mixed-effects model). (h) Line plot for the log2-normalized bulk RNA-seq expression values of CXCR4 are shown. The p-value was calculated with the two-sided Wald-test in DESeq2 and corrected for multiple testing using Benjamini-Hochberg method. (i) Line plot showing the proportion of CXCR4-positive plasma cells in the focal lesion compared to the random sample per patient (n=8 patients with bulk RNA-seq data) based on immunohistochemistry staining. The p-value was calculated with two-sided Wilcoxon signed rank test. (j) Boxplot for the proportion of mesenchymal stroma cells (MSCs) according to flow cytometry for 8 patients with paired RBM/FL. Proportions were calculated based on the CD138-depleted fractions. The p-value was calculated with two-sided Wilcoxon signed rank test. The boxplots show the median and the interquartile range, while the upper and lower whiskers show the highest and lowest value. Source data are provided as a Source Data file.

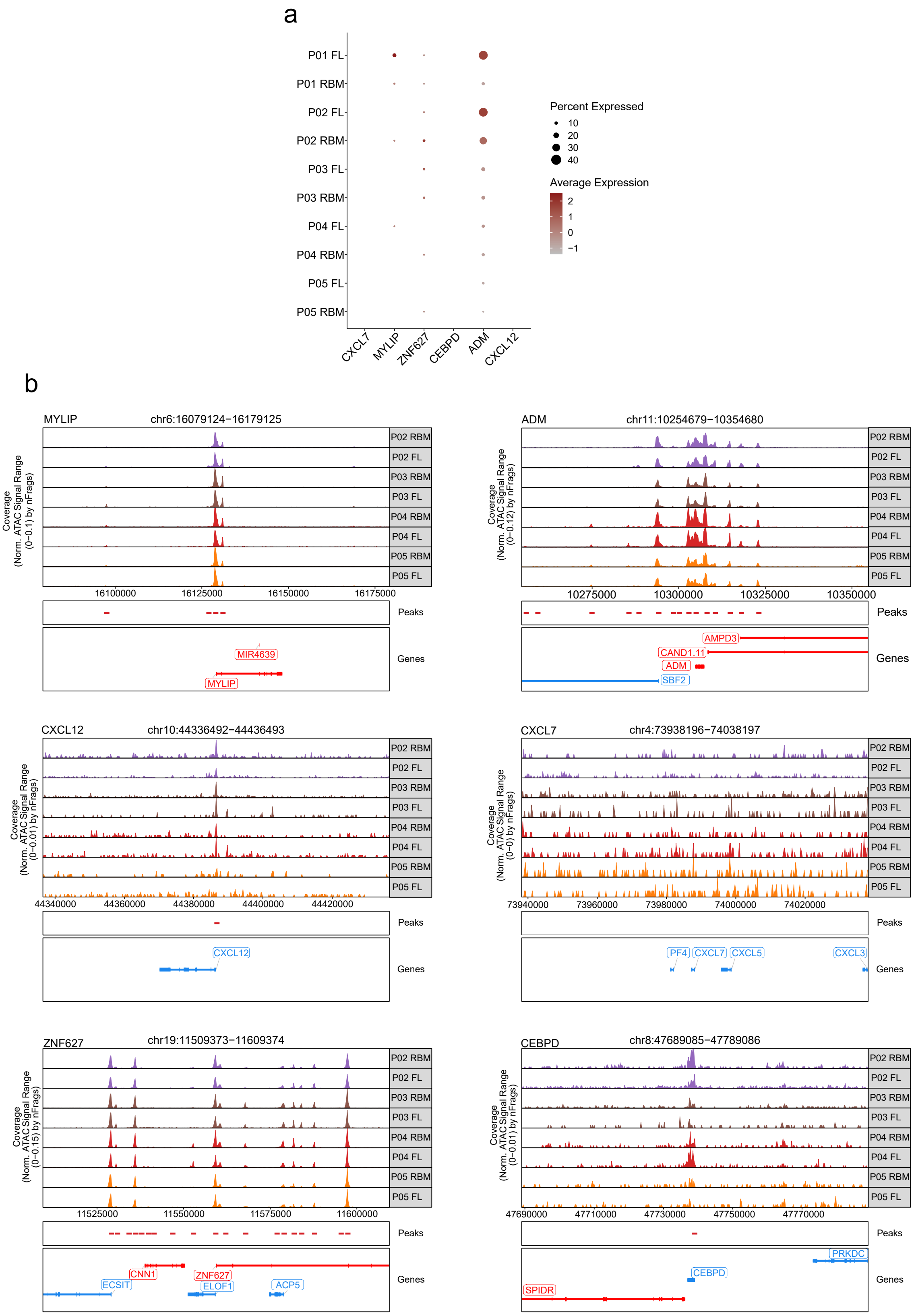

Suppl. Fig. 6: Detection of the 6 top differentially expressed genes between focal lesion and random sample in single cell data. (a) scRNA-seq expression of the 6 validated genes from the bulk expression analysis (RNA-seq and microarray dataset (University of Arkansas for Medical Sciences)) in the 5 paired FL/RBM samples. (b) Aggregated pseudo-bulk scATAC-seq tracks of the 6 top differentially expressed genes *MYLIP*, *ADM*, *CXCL12*, *CXCL7* (*PPBP*), *ZNF627* and *CEBPD* for all 4 patients. Detected peaks are marked in red.

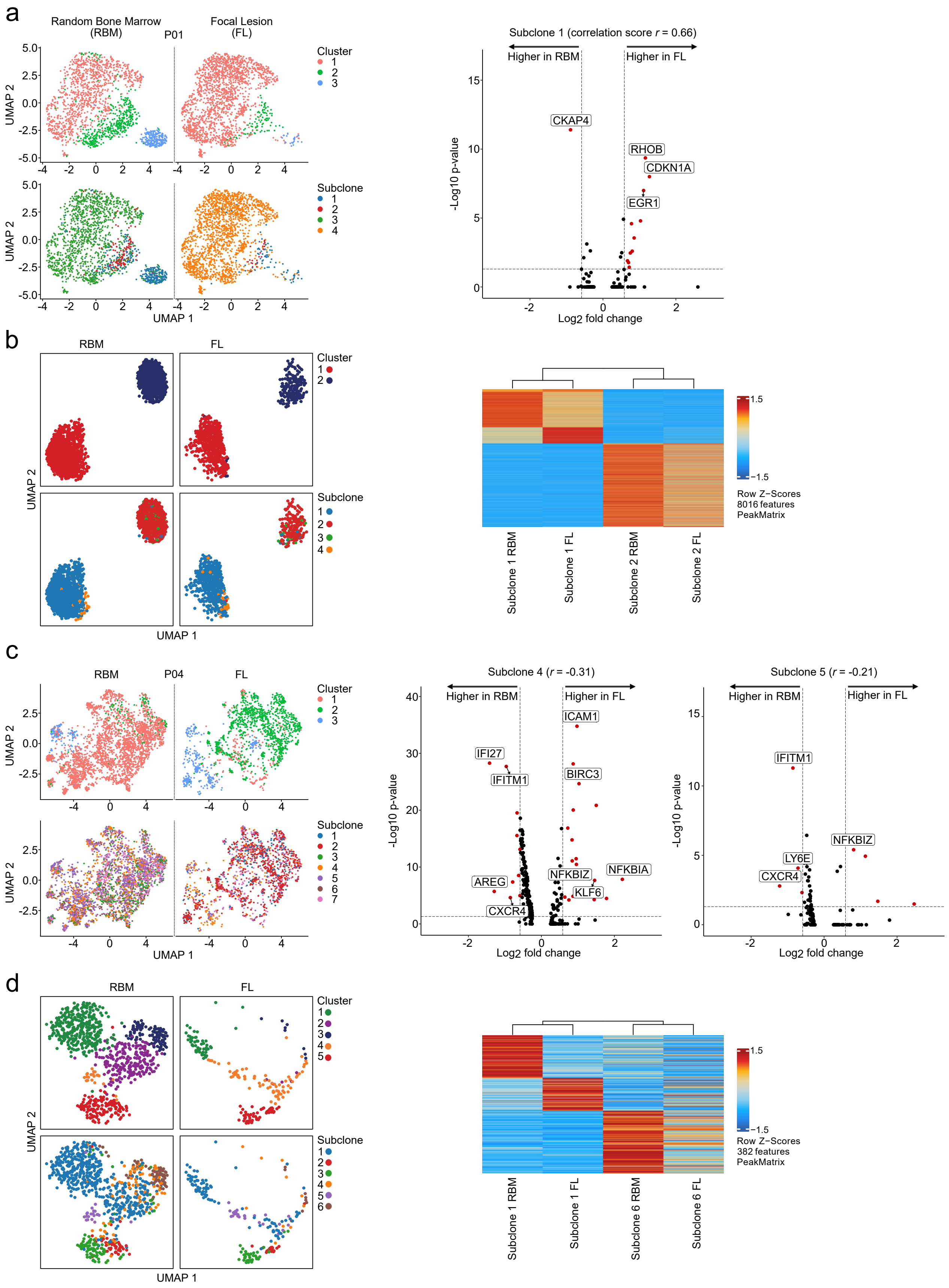

(legend on next page)

Suppl. Fig. 7: Spatial transcriptional and epigenetic plasticity in tumor subclones. (a) *Left panel:* Uniform Manifold Approximation and Projection (UMAP) of transcriptional clusters and CNA-subclones in patient P01 split by BM-site (random bone marrow (RBM) and focal lesion (FL)). *Right panel:* Volcano Plots of the differentially expressed genes between FL and RBM of subclones with >50 cells at both BM-sites. Red points indicate genes with an adjusted p-value<0.05 and 1.5-fold expression difference between both sites. (b) Single-cell ATAC-seq data for patient P05 as an example for very similar chromatin accessibility profiles of genetically identical subclones in paired samples. *Left panel*: chromatin accessibility clusters and copy number aberration (CNA)-defined subclones *Right panel*: scATAC-seq heatmap of differential accessibility peaks across subclones. Color indicates the column Z-score of normalized peak accessibility. (c) *Left panel:* Uniform Manifold Approximation and Projection (UMAP) of transcriptional clusters and CNA-subclones in patient P04 split by BM-site (random bone marrow (RBM) and focal lesion (FL)). *Right panel:* Volcano Plots of the differentially expressed genes between FL and RBM of subclones with >50 cells at both BM-sites. Red points indicate genes with an adjusted p-value of <0.05 and 1.5-fold expression difference between both sites. (d) In (d) the same plots as in (b) are shown for patient P03 as an example for pronounced differences between genetically identical subclones at different bone marrow sites. The two subclones 1 and 6 were assigned to different chromatin accessibility clusters at the FL and the RBM, suggesting differential chromatin accessibility profiles of the same subclone at different bone marrow sites. Of note, the cell number of subclone 6 in the FL was <50 cells. P-values were calculated based on two-sided Wilcoxon rank sum test and Benjamini-Hochberg adjustment.

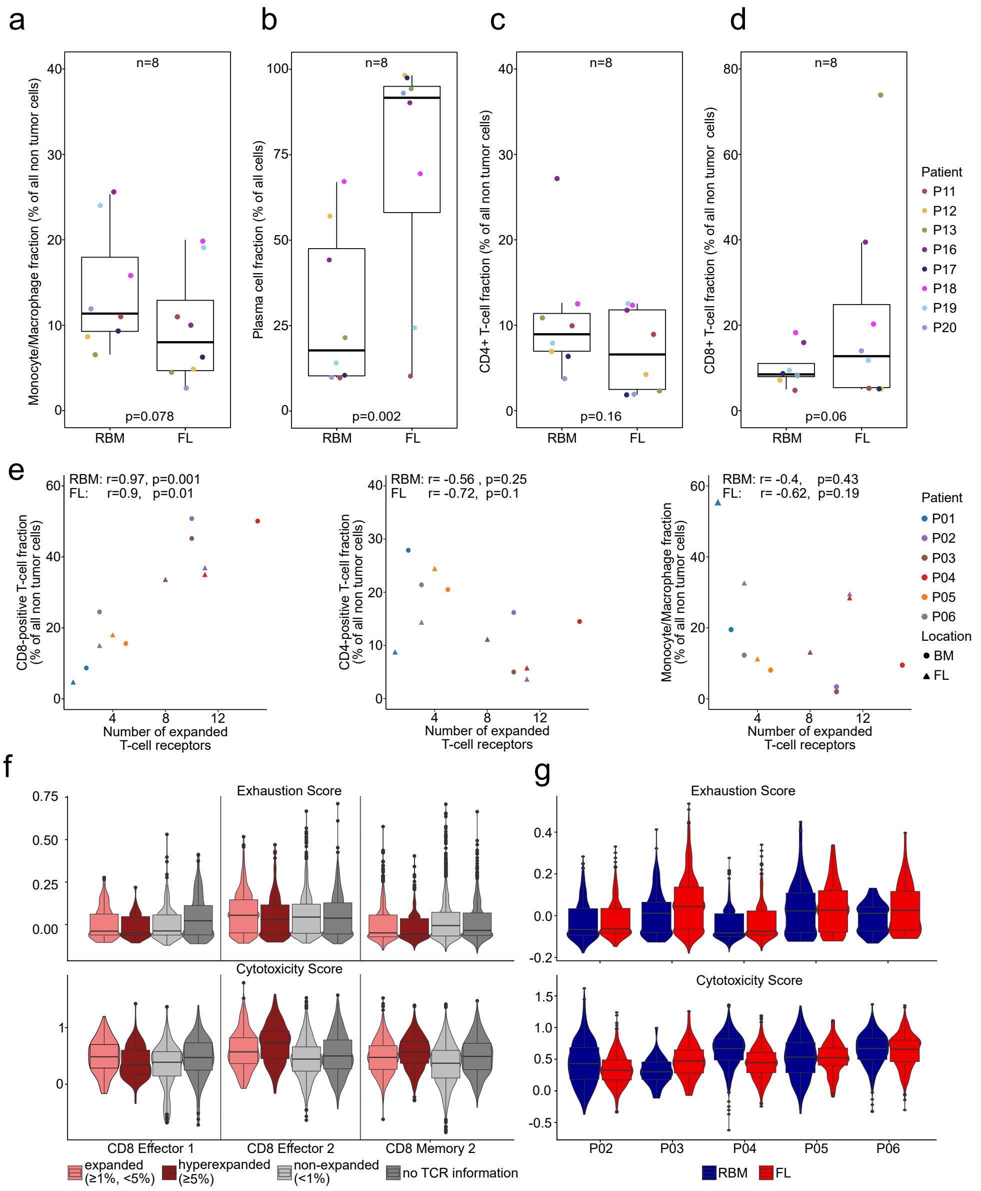

Suppl. Fig. 8: Spatial heterogeneity in the microenvironment and T-cell receptor repertoire. (a) Boxplot for the proportion of macrophages according to flow cytometry for 8 patients with paired RBM/FL samples. Proportions were calculated based on the CD138-depleted fractions. (b) Boxplot of the plasma cell infiltration of 8 patients with paired RBM/FL samples according to flow cytometry. (c,d) Boxplots for the proportion of CD4- (c) and CD8-positive (d) T-cells according to flow cytometry for 8 patients with paired RBM/FL samples. Proportions were calculated based on the CD138-depleted fractions. All p-values were calculated with two-sided Wilcoxon signed rank test. (e) Association between the number of expanded T-cell clones (x-axis) and the proportion of CD8- (left), CD4-positive T-cells (middle) and macrophages (right) (y-axis) for all 6 patients with scRNA-seq data. Each sample was plotted separately and colored by patient and location. The correlation was separately tested for focal lesion and random bone marrow samples using Pearson correlation. (f) Recently published T-cell exhaustion and cytotoxicity scores[2] were calculated for CD8 effector 1 and 2 as well as CD8 memory 2 T-cells for all patients with TME scRNA-seq data combined. No significant difference could be observed between (hyper-)expanded and non-expanded T-cells. (NA: no T-cell receptor information available). (g) T-cell exhaustion and cytotoxicity scores for expanded T-cells (≥1%) splitted by sample origin for all patients with TME scRNA-seq data combined. Cell numbers are provided in the Source Data (f-g). The boxplots show the median and the interquartile range, while the upper and lower whiskers show the highest and lowest value. Source data are provided as a Source Data file.

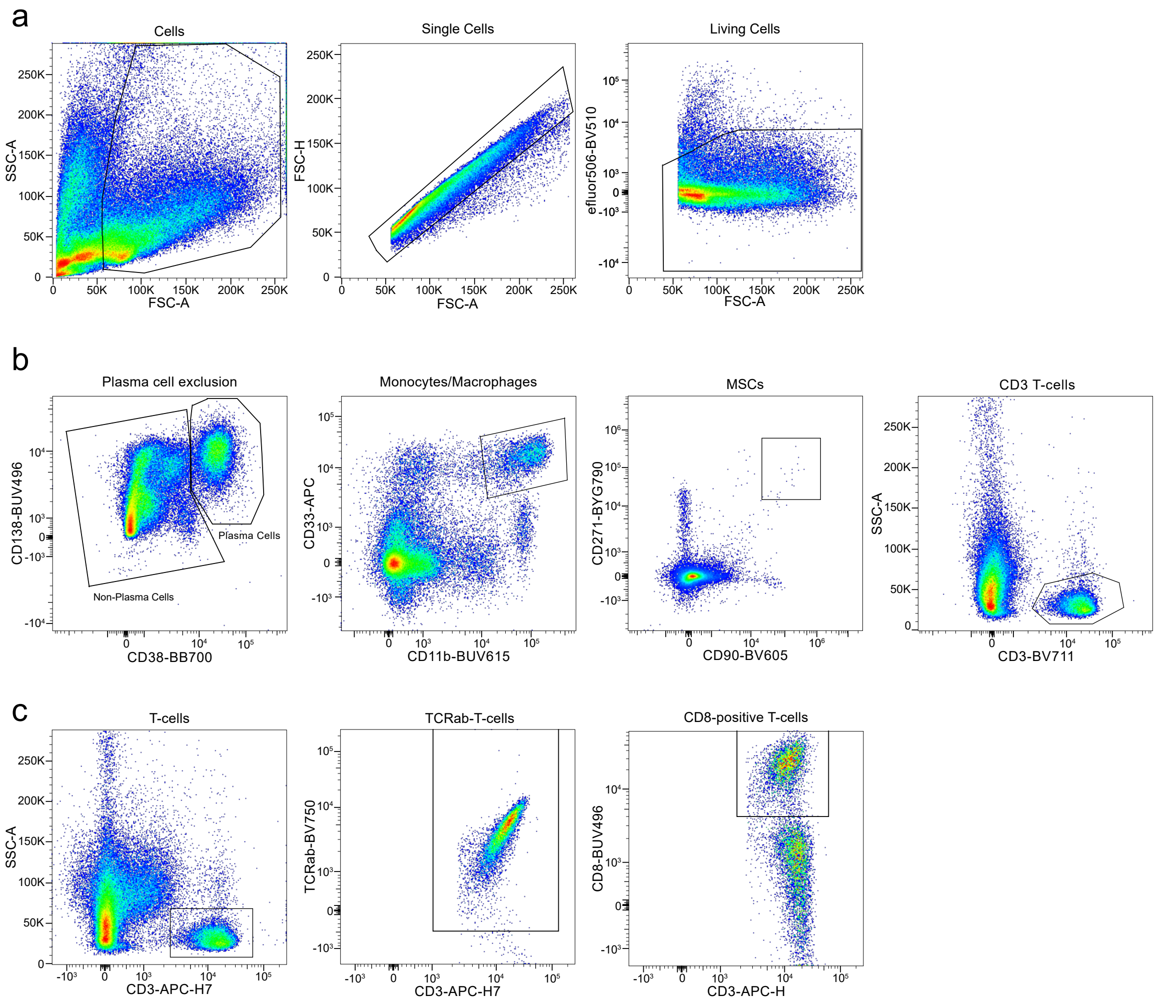

Suppl. Fig. 9: Gating strategy flow cytometry analysis. (a) Gating strategy to identify living cells for downstream analysis. (b) Plasma cells were identified as CD38+ CD138+ (see Suppl. Fig. 8b) and were excluded from further gating. Monocytes/macrophages were defined as CD11b+ CD33+ (see Suppl. Fig. 8a), mesenchymal stromal cells as CD90+ CD271+ (see Suppl. Fig. 5j) and T-cells as CD3+. (c) T-cell focus. CD8+ T-cells were defined as CD3+, TCRab+ and CD8+ (see Suppl. Fig. 8c+d). For the analysis of (b) and (c) the sample was analyzed in two tubes: one tube for a general cell type annotation and the second one to define subsets of T-cells.

Supplementary Tables

Suppl. Table 1: Driver mutations of all samples included in this study.

PID	Group	Driver mutations*
P01	Shared	-
P01	Enriched RBM	-
P01	Enriched FL	*KRAS*^{G35T}
P02	Shared	*PTPN11*^{C1520A}
P02	Enriched RBM	*KRAS*^{G35A}, *NRAS*^{G38A}
P02	Enriched FL	*KRAS*^{G38A}, *CDKN2C*$^{T365C/del}$
P03	Shared	*KMT2B*^{G413A}, *TP53*^{T425A}, *RB1*$^{del/del}$, *RASA2*$^{del/del}$
P03	Enriched RBM	-
P03	Enriched FL	-
P04	Shared	-
P04	Enriched RBM	*KRAS*^{A183C}
P04	Enriched FL	*CYLD*$^{G1466T/del}$
P05	Shared	-
P05	Enriched RBM	-
P05	Enriched FL	-
P07	Shared	*NRAS*^{G35A}
P07	Enriched RBM	-
P07	Enriched FL	-
P08	Shared	-
P08	Enriched RBM	*TP53*$^{C817T/del}$
P08	Enriched FL	*TP53*$^{G538A/del}$, *RB1*$^{del/del}$, *CYLD*$^{del/del}$
P09	Shared	*NRAS*^{C181A}, *PRKD2*^{C1676T}, *IRF4*^{A368G}
P09	Enriched RBM	-
P09	Enriched FL	-
P10	Shared	*KRAS* ^{A183C}
P10	Enriched RBM	-
P10	Enriched FL	-
P11	Shared	*SAMHD1*^{G371A}
P11	Enriched RBM	-
P11	Enriched FL	-
P12	Shared	-
P12	Enriched RBM	-
P12	Enriched FL	-
P13	Shared	*LTB*^{G157A}
P13	Enriched RBM	*KRAS*^{G436A}
P13	Enriched FL	*KRAS*^{G35C}
P14	Shared	*FAM46C*^{C1015T}
P14	Enriched RBM	-
P14	Enriched FL	*KRAS*^{A183C}
P15	Shared	*TRAF2*^{G1366T}, *TP53*^{G839A}
P15	Enriched RBM	-
P15	Enriched FL	-
P16	Shared	*NRAS*^{A182G}
P16	Enriched RBM	-
P16	Enriched FL	-
`P17	Shared	-
P17	Enriched RBM	-
P17	Enriched FL	-

RBM: random bone marrow; FL: focal lesion;
*Driver mutations according to Walker et al.[1]
Enriched means unshared or threefold difference in the cancer clonal fractions between paired samples.

Suppl. Table 2: Details of the linear mixed-effects model used for immunohistochemistry data.

Coeffcients for CD68	Estimate	Standard Error	t-value	*p*-value
(Intercept)	41.04	2.25	18.22	<0.0001
Inf2	-16.30	2.46	-6.62	<0.0001
Inf3	-29.06	2.24	-12.95	<0.0001
Inf4	-40.25	1.93	-20.87	<0.0001
Sample RBM	8.23	1.20	6.85	<0.0001
	Correlation between predictors*: *r*=0.29			
**Coeffcients for CD4**	**Estimate**	**Standard Error**	**t-value**	**p-value**
(Intercept)	22.99	2.02	11.39	<0.0001
Inf2	-8.70	2.38	-3.65	0.0003
Inf3	-16.99	2.16	-7.86	<0.0001
Inf4	-21.49	1.95	-11.03	<0.0001
Sample RBM	-0.94	1.05	-0.90	3.68 E-01
	Correlation between predictors*: *r*=0.21			
**Coeffcients for CD8**	**Estimate**	**Standard Error**	**t-value**	**p-value**
(Intercept)	17.88	2.23	8.00	<0.0001
Inf2	0.31	2.57	0.12	0.91
Inf3	-4.92	2.23	-2.21	0.027
Inf4	-14.99	2.02	-7.41	<0.0001
Sample RBM	3.86	1.138	3.39	0.0006
	Correlation between predictors*: *r*=0.19			

Inf2: 26-50% Plasma cell infiltration, Inf3:51-75% plasma cell infiltration, Inf4: 76-100% Plasma-cell infiltration. RBM: random bone marrow, VIF<1.1; *for the correlation analysis numerical values for predictors were used.

Suppl. Table 3: Overview of samples and performed analyses per patient.

PID	WGS	RNA bulk	scRNA tumor	scRNA TME	scATAC tumor	FACS	ELISA CXCL12	IHC Ki67/CXCL12/ CXCR4	IHC Monocytes/ T-cells
P01	green	green	green	green	grey	grey	grey	green	green
P02	green	green	green	green	green	grey	grey	green	green
P03	green	green	green	green	green	grey	grey	green	green
P04	green	green	green	green	green	grey	green	green	green
P05	green	green	green	green	green	grey	green	green	green
P06	grey	grey	grey	green	grey	grey	grey	grey	green
P07	green	green	grey	grey	grey	grey	grey	grey	grey
P08	green	grey	grey	grey	grey	grey	grey	grey	grey
P09	green	grey	grey	grey	grey	grey	grey	grey	grey
P10	green	grey	grey	grey	grey	grey	grey	grey	grey
P11	green	green	grey	grey	grey	green	grey	green	green
P12	green	green	grey	grey	grey	green	green	green	green
P13	green	green	grey	grey	grey	green	green	green	green
P14	green	grey	grey	grey	grey	grey	grey	grey	grey
P15	green	grey	grey	grey	grey	grey	grey	grey	green
P16	green	green	grey	grey	grey	green	green	grey	grey
P17	green	green	grey	grey	grey	green	green	grey	grey
P18	grey	grey	grey	grey	grey	green	grey	grey	grey
P19	grey	grey	grey	grey	grey	green	grey	grey	grey
P20	grey	grey	grey	grey	grey	green	grey	grey	grey
P21	grey	grey	grey	grey	grey	grey	grey	grey	green
P22	grey	grey	grey	grey	grey	grey	grey	grey	green
P23	grey	grey	grey	grey	grey	grey	grey	grey	green
P24	grey	grey	grey	grey	grey	grey	grey	grey	green
P25	grey	grey	grey	grey	grey	grey	grey	grey	green
P26	grey	grey	grey	grey	grey	grey	grey	grey	green
P27	grey	grey	grey	grey	grey	grey	grey	grey	green
P28	grey	grey	grey	grey	grey	grey	grey	grey	green
P29	grey	grey	grey	grey	grey	grey	grey	grey	green
P30	grey	grey	grey	grey	grey	grey	grey	grey	green
P31	grey	grey	grey	grey	grey	grey	grey	grey	green

green: analysis performed with the paired random and focal lesion sample, grey: no paired samples available

Suppl. Table 4: Patient characteristics

PID	Age at Diagnosis	R-ISS	FISH-Risk	IG Type	Origin of focal lesion sample	Type of investigated lesion
P01	<65	I	standard	BJ kappa	left iliac crest	intramedullary
P02	<65	III	standard	IgG kappa	right iliac crest	intramedullary
P03	<65	II	standard	IgG kappa	right iliac crest	intramedullary
P04	<65	II	standard	IgG kappa	left iliac crest	intramedullary
P05	<65	I	standard	Light chain lambda	right iliac crest	intramedullary
P06	<65	I	standard	IgA kappa	left iliac crest	intramedullary
P07	<65	I	standard	IgA kappa	right iliac crest	intramedullary
P08	<65	II	amp(1q21) del(17p)	IgA kappa	left iliac crest	intramedullary
P09	>65	II	standard	IgA lambda	right humerus (surgical resection)	paramedullary
P10	>65	I	standard	BJ lambda	right iliac crest	intramedullary
P11	<65	I	standard	IgG kappa	left iliac crest	intramedullary
P12	<65	I	standard	BJ lambda	3rd cervical vertebra (surgical resection)	paramedullary
P13	>65	NA	gain(1q21)	IgA kappa	2nd lumbal vertebra (surgical resection)	paramedullary
P14	<65	I	gain(1q21)	IgG kappa	left iliac crest	intramedullary
P15	>65	III	standard	IgG kappa	left iliac crest	intramedullary
P16	<65	I	standard	IgG kappa	1st lumbal vertebra (surgical resection)	paramedullary
P17	>65	I	gain(1q21)	BJ kappa	6/7th cervical vertebra (surgical resection)	paramedullary
P18	<65	II	standard	IgG kappa	left iliac crest	intramedullary
P19	<65	I	gain(1q21)	IgG kappa	left iliac crest	intramedullary
P20	>65	II	standard	IgG kappa	2nd lumbal vertebra (surgical resection)	paramedullary
P21	<65	III	t(4;14)+ gain(1q21)	IgA kappa	5. thoracic vertebra	paramedullary
P22	>65	ISS III	unknown	IgG kappa	Left proximal femur (surgical resection)	intramedullary
P23	<65	I	standard	IgG kappa	right sacrum	intramedullary
P24	>65	I	standard	BJ lambda	right pubis	intramedullary
P25	<65	III	gain(1q21)	IgG lambda	left iliac crest	paramedullary
P26	>65	ISS I	unknown	Light chain lambda	left ulna	intramedullary
P27	<65	II	t(4;14)+ gain(1q21)	IgG lambda	left ilium	intramedullary
P28	<65	I	standard	IgG kappa	right femur neck (surgical resection)	intramedullary
P29	<65	ISS II	unknown	IgG lambda	right ilium	intramedullary
P30	>65	II	standard	IgG kappa	left ilium	intramedullary
P31	<65	II	standard	BJ lambda	left humerus	intramedullary

PID: patient identifier; R-ISS: revised international staging system; FISH-Risk (random iliac crest sample): gain(1q), del(17p), t(4;14) and t(14;16) were considered as high-risk markers; IG: Immunoglobulin; BJ: Bence Jones

Suppl. Table 5: Sample processing and purity.

	Random sample			Focal Lesion		
PID	Sample type	Sorting Method	Purity (WGS)*	Sample type	Sorting method	Purity (WGS)*
P01	Aspirate	Robosep	0.9	Aspirate	Robosep	0.8
P02	Aspirate	Robosep	0.96	Aspirate	Robosep	0.92
P03	Aspirate	Robosep	1	Aspirate	Robosep	1
P04	Aspirate	Robosep	0.92	Aspirate	Robosep	0.98
P05	Aspirate	Robosep	0.78	Aspirate	Robosep	0.8
P06	Aspirate	Robosep	NA	Aspirate	Robosep	NA
P07	Aspirate	Robosep	0.98	Aspirate	Robosep	0.86
P08	Aspirate	Robosep	0.9	Aspirate	Robosep	0.84
P09	Aspirate	Robosep	0.96	Surgical resection	fresh-frozen tissue, no sort	0.84
P10	Aspirate	Robosep	0.82	Aspirate	Robosep	0.92
P11	Aspirate	Robosep	0.52	Aspirate	Robosep	0.9
P12	Aspirate	Robosep	0.98	Surgical resection	FACSAria	0.98
P13	Aspirate	Robosep	0.88	Surgical resection	FACSAria	0.98
P14	Aspirate	Robosep	0.98	Aspirate	Robosep	0.91
P15	Aspirate	Robosep	1	Aspirate	Robosep	1
P16	Aspirate	Robosep	0.86	Surgical resection	FACSAria	0.98
P17	Aspirate	Robosep	0.7	Surgical resection	FACSAria	0.98

WGS: whole genome sequencing
* The tumor purity was estimated based on histograms for the variant allele frequency (VAF) of mutations according to whole genome sequencing

Suppl. Table 6: Antibodies used for flow cytometry or immuohistochemistry stainings

Antibody	Tube	Method	Conjugate	Clone	Dilution	Source	Cat#	Validated*
Mouse anti-human CD3	1	Flow cytometry	BV711	SK7	1:100	BD Biosciences	740832	yes
Rat anti-human CD11b	1	Flow cytometry	BUV615	M1/70	1:50	BD Biosciences	751140	yes
Mouse anti-human CD33	1	Flow cytometry	APC	WM53	1:20	BD Biosciences	551378	yes
Mouse anti-human CD34	1	Flow cytometry	PE	581	1:5	BD Biosciences	555822	yes
Mouse anti-human CD38	1	Flow cytometry	PerCPCy5.5	HIT2	1:100	BD Biosciences	551400	yes
Mouse anti-human CD45RA	1	Flow cytometry	BB515	HI100	1:100	BD Biosciences	564552	yes
Mouse anti-human CD90	1	Flow cytometry	BV605	5E10	1:50	BD Biosciences	747750	yes
Mouse anti-human CD138	1	Flow cytometry	BUV496	MI15	1:50	BD Biosciences	749874	yes
Mouse anti-human CD271	1	Flow cytometry	PE-Cy7	ME20.4-1.H4	1:50	Miltenyi-Biotech	345110	yes
Mouse anti-human CD3	2	Flow cytometry	APC-H7	SK7	1:20	BD Biosciences	560176	yes
Mouse anti-human CD4	2	Flow cytometry	BUV395	SK3	1:100	BD Biosciences	563550	yes
Mouse anti-human CD8	2	Flow cytometry	BUV496	SK1	1:100	BD Biosciences	741199	yes
Mouse anti-human TCRαβ	2	Flow cytometry	BV750	IP26	1:50	BD Biosciences	747180	yes
Rabbit anti-human CD4	-	IHC	-	SP35	Ready-to-use	Roche Ventana	790-4423	yes
Rabbit anti-human CD8	-	IHC	-	SP57	Ready-to-use	Roche Ventana	790-4460	yes
Mouse anti-human CD68	-	IHC	-	KP-1	Ready-to-use	Roche Ventana	790-2931	yes
Mouse anti-human CD138	-	IHC	-	B-A38	Ready-to-use	Roche Ventana	760-4248	yes
Rabbit anti-human CXCL7	-	IHC	-	poly-clonal	1:20	LSBio	LS-B7394	yes
Rabbit anti-human CXCL12	-	IHC	-	poly-clonal	1:40	LSBio	LS-B7489	yes
Mouse anti-human CXCR4	-	IHC	-	12G5	1:50	LSBio	LS-B1986	yes
Rabbit anti-human Ki67	-	IHC	-	30-9	Ready-to-use	Roche Ventana	790-4286	yes
Mouse anti-human MUM1	-	IHC	-	MUM1P	1:50	Agilent DAKO	M7259	Yes

*Validated for the respective application according to the manufacturer's technical data sheet.

Suppl. Table 7: Number of cells in scRNA-seq before and after quality control

PID	Sample	# cells before filtering	# cells after filtering
P01	RBM_CD138pos	4307	2284
P01	RBM_CD138neg	2510	1409
P01	FL_CD138pos	3420	2313
P01	FL_CD138neg	3367	2221
P02	RBM_CD138pos	6889	4606
P02	RBM_CD138neg	3430	2806
P02	FL_CD138pos	8505	6114
P02	FL_CD138neg	5948	3629
P03	RBM_CD138pos	6412	3557
P03	RBM_CD138neg	6391	3951
P03	FL_CD138pos	2348	1397
P03	FL_CD138neg	7812	5461
P04	RBM_CD138pos	4967	4129
P04	RBM_CD138neg	7816	5936
P04	FL_CD138pos	2582	2197
P04	FL_CD138neg	5385	3420
P05	RBM_CD138pos	3537	1972
P05	RBM_CD138neg	6691	5132
P05	FL_CD138pos	2948	1390
P05	FL_CD138neg	5224	3777
P06	RBM_CD138neg	4971	3610
P06	FL_CD138neg	4684	3320

RBM: random bone marrow; FL: focal lesion; CD138pos: CD138-enriched cells; CD138neg: CD138-depleted cells (negative fraction of CD138 sort)

Suppl. Table 8: Number of cells in scATAC-seq before and after quality control

PID	Sample	# cells before filtering	# cells after filtering
P02	RBM_CD138pos	6110	376
P02	FL_CD138pos	985	594
P03	RBM_CD138pos	914	839
P03	FL_CD138pos	201	196
P04	RBM_CD138pos	1326	1215
P04	FL_CD138pos	623	555
P05	RBM_CD138pos	3137	2920
P05	FL_CD138pos	2395	2210

RBM: random bone marrow; FL: focal lesion; CD138pos: CD138-enriched cells

Suppl. Table 9: Chromosomal regions used for supervised clustering of single-cell sequencing data

PID	Sample	Regions with subclonal CNAs [in MBP]
P01	RBM	4 subclone solution by InferCNV was confirmed by WGS, no further clustering required
P01	FL	4 subclone solution by InferCNV was confirmed by WGS, no further clustering required
P02	RBM	round1: chr6p (<25); round2: (chr6p+) chr19, (chr6p-) chr19
P02	FL	round1: chr6p (<20); round2 (chr6p-): chr20; round3 (chr20-): chr19; round4 (chr19+): chr4 (100-150)
P03	RBM	round1: chr11; round2: chr21; round3: chr4 (<105); round4: chr16 (>48)
P03	FL	round1: chr11; round2: chr21; round3: chr4 (<105); round4: chr16 (>48)
P04	RBM	round1: chr14+19q (cut into 6 clones); round2 Clone1-3 : chr13 (50-110), Clone4-6 chr13&chr14
P04	FL	round1: chr14+19q (cut into 6clones); round2 Clone1,4 : chr13 (>45); Clone2,3,5,6 chr13+chr14
P05	RBM	round1: chr8(<40); round2: chr7 (>100); round3: chr6 (<40)
P05	FL	round1: chr8(<40); round2: chr7 (>100); round3: chr6 (<40)

RBM: random bone marrow; FL: focal lesion; CNA: copy number aberration; Mbp: Million base pairs; WGS: whole genome sequencing

Example for the supervised clustering approach (P05, presented in Fig. 2a):

(1) P05 was first clustered based on chr8p (<40 Mbp)
→ Cells with del(8p): Subclone1
(2) Cells without del(8p) were further clustered for chr7q (>100 Mbp)
→ Cells with gain(7q): Subclone2
(3) Cells without gain(7q) were then clustered for chr6p (<40 Mbp)
→ Cells with gain(6p): Subclone3
→ Cells without gain(6p): Subclone4

Supplementary References

1 Walker, B. A. *et al.* Identification of novel mutational drivers reveals oncogene dependencies in multiple myeloma. *Blood* **132**, 587-597, doi:10.1182/blood-2018-03-840132 (2018).

2 Yang, R. *et al.* Distinct epigenetic features of tumor-reactive CD8+ T cells in colorectal cancer patients revealed by genome-wide DNA methylation analysis. *Genome Biol* **21**, 2, doi:10.1186/s13059-019-1921-y (2019).
