# Supplementary material for: Resolving the spatial architecture of myeloma and its microenvironment at the single-cell level
Source: Nat Commun. 2023 Aug 17;14:5011. doi: 10.1038/s41467-023-40584-4 (PMC10435504; doi:10.1038/s41467-023-40584-4)
Supplement: Supplementary file 3 — Description of Additional Supplementary Files [file 41467_2023_40584_MOESM3_ESM.pdf]

Description of Additional Supplementary Files

Supplementary Data 1: Copy number aberration differences between paired samples (Chr: chromosome; CN: copy number; RBM: random bone marrow; LOH: loss of heterozygosity).

Supplementary Data 2: Differential expression of bulk RNA-seq data between paired focal lesion and RBM samples of 11 patients (adjusted *p*-value<=0.05 and 1.5-fold enrichment) and comparison with a dataset from the University of Arkansas for Medical Sciences (UAMS) dataset (n=250 patients). All genes which were significant in both datasets are marked in bold. P-values were calculated based on two-sided Wald test in DESeq2 and corrected for multiple testing using Benjamini-Hochberg method. lfc: Log2FoldChange; SE: standard error; n.s. non-significant; RBM: random bone marrow

Supplementary Data 3: Association of *CXCL12*, *CXCL7* and *CD74* expression with clinical parameters at baseline in a cohort of 653 newly diagnosed myeloma patients[1]. P-values were calculated based on t-test (PC-Infiltration) or chi-square test.

Supplementary Data 4: Differential expression of the patients P01, P03, P04 and P05 between tumor subclones at different BM-sites (see Excel file).

- Sheet1: Differentially expressed genes of P05 focal lesion vs RBM subclone 1
- Sheet2: Differentially expressed genes of P05 focal lesion vs RBM subclone 2
- Sheet3: Differentially expressed genes of P01 focal lesion vs RBM subclone 1
- Sheet4: Differentially expressed genes of P04 focal lesion vs RBM subclone 4
- Sheet5: Differentially expressed genes of P04 focal lesion vs RBM subclone 5
- Sheet6: Differentially expressed genes of P03 focal lesion vs RBM subclone 1
- Sheet7: Differentially expressed genes of P03 focal lesion vs RBM subclone 6
- Sheet8: Pathway enrichment analysis with the differentially expressed genes of subclone 1, respectively subclone6, separately for focal lesion and RBM (HALLMARK pathways)
- Sheet9: Differentially expressed genes of P01 over all tumor subclones

Supplementary Data 5: Proportions of immune cell types in the tumor microenvironment (CD138-depleted fraction) in paired focal lesion (FL) and RBM samples. P-values were calculated based on two-sided Wilcoxon signed-rank test.

Supplementary Data 6: Overview of T-cell receptor clones in paired samples.
RBM: random bone marrow; FL: focal lesion
*database query[2,3] with the CDR3 sequences of the expanded T-cell clones to test for known antigen specificities

Supplementary Data 7: Overview of corresponding T-cell subtypes for the expanded T-cell receptor clones.

Supplementary Data 8: Size and anatomic location for the analyzed focal lesion and the 3 largest focal lesions per patient. FL: focal lesion (see Excel file, RBM: random bone marrow; FL: focal lesion)

Supplementary References

1 Weinhold, N. *et al.* Chromosome 1q21 abnormalities refine outcome prediction in patients with multiple myeloma - a meta-analysis of 2,596 trial patients. *Haematologica* **106**, 2754-2758, doi:10.3324/haematol.2021.278888 (2021).

2 Bagaev, D. V. *et al.* VDJdb in 2019: database extension, new analysis infrastructure and a T-cell receptor motif compendium. *Nucleic Acids Res* **48**, D1057-d1062, doi:10.1093/nar/gkz874 (2020).

3 Tickotsky, N., Sagiv, T., Prilusky, J., Shifrut, E. & Friedman, N. McPAS-TCR: a manually curated catalogue of pathology-associated T cell receptor sequences. *Bioinformatics* **33**, 2924-2929, doi:10.1093/bioinformatics/btx286 (2017).
