# Supplementary material for: Resolving the spatial architecture of myeloma and its microenvironment at the single-cell level
Source: Nat Commun. 2023 Aug 17;14:5011. doi: 10.1038/s41467-023-40584-4 (PMC10435504; doi:10.1038/s41467-023-40584-4)
Supplement: Supplementary file 12 — Reporting Summary [file 41467_2023_40584_MOESM12_ESM.pdf]

nature portfolio

Corresponding author(s): Niels Weinhold

Last updated by author(s): Jul 26, 2023

Reporting Summary

Nature Portfolio wishes to improve the reproducibility of the work that we publish. This form provides structure for consistency and transparency in reporting. For further information on Nature Portfolio policies, see our Editorial Policies and the Editorial Policy Checklist.

Statistics

For all statistical analyses, confirm that the following items are present in the figure legend, table legend, main text, or Methods section.

n/a	Confirmed	
☐	☒	The exact sample size (n) for each experimental group/condition, given as a discrete number and unit of measurement
☐	☒	A statement on whether measurements were taken from distinct samples or whether the same sample was measured repeatedly
☐	☒	The statistical test(s) used AND whether they are one- or two-sided<br>*Only common tests should be described solely by name; describe more complex techniques in the Methods section.*
☐	☒	A description of all covariates tested
☐	☒	A description of any assumptions or corrections, such as tests of normality and adjustment for multiple comparisons
☐	☒	A full description of the statistical parameters including central tendency (e.g. means) or other basic estimates (e.g. regression coefficient) AND variation (e.g. standard deviation) or associated estimates of uncertainty (e.g. confidence intervals)
☐	☒	For null hypothesis testing, the test statistic (e.g. F, t, r) with confidence intervals, effect sizes, degrees of freedom and P value noted<br>*Give P values as exact values whenever suitable.*
☒	☐	For Bayesian analysis, information on the choice of priors and Markov chain Monte Carlo settings
☒	☐	For hierarchical and complex designs, identification of the appropriate level for tests and full reporting of outcomes
☐	☒	Estimates of effect sizes (e.g. Cohen's d, Pearson's r), indicating how they were calculated

Our web collection on statistics for biologists contains articles on many of the points above.

Software and code

Policy information about availability of computer code

Data collection

Patients: This study was approved by the Heidelberg University Medical Faculty ethics review board (S278-13) and complied with all relevant ethical regulations. Signed written informed consent for sample procurement and processing in accordance with the Declaration of Helsinki was obtained for all cases included in this study. Study subjects did not receive compensation for participation in the study. For the comparison of paired samples, we included 31 Caucasians with newly diagnosed myeloma and accessible focal lesions. Sex was not considered in the initial study design due to the small number of eligible patients. All patients fulfilled the International Myeloma Working Group (IMWG) criteria for treatment. CD138-enriched MM cells for molecular analyses were available from 17 of these patients. Patient clinical characteristics, the origin of samples and the analyses, which were performed with the respective samples, are shown in Fig. 1b and Suppl. Table 3+4. For correlation analyses between gene expression and patient characteristics as well as outcome, we included 653 newly diagnosed patients from two phase III clinical studies (GMMG HD4 and MM5 trials) with available gene expression profiling data.
Datasets: Whole genome sequencing libraries were prepared with the Illumina TruSeq Nano DNA kit and sequenced on a HiSeq X (paired-end 150 bp) to an average coverage of 85x for tumor and 43x for germline control samples, respectively.
RNA-Seq libraries were prepared using the Illumina TruSeq stranded mRNA kit and sequenced on the Illumina NovaSeq 6000 PE 100 S1 platform.
GEP of CD138-enriched BM plasma cells was performed using Affymetrix U133Plus2.0 microarrays (Santa Clara, CA) according to the manufacturer's instructions.
For CD138+ and CD138- BMMCs single-cell RNA plus B and T-cell receptor sequencing were performed using the Chromium Next GEM Single Cell 5' Reagent Kit v1.1 and the V(D)J Reagent Kit v1.1 according to the manufacturer's protocol (14,000 cells per channel). Generated gene expression libraries were paired-end sequenced on the NovaSeq 6000 S2. Generated V(D)J libraries were paired-end sequenced on the NextSeq 550.
For scATAC-seq, viably frozen CD138+ BMMCs were thawed and washed once with 1x PBS. Cell pellets were carefully resuspended in an ice-cold NP-40 lysis buffer (10 mM Tris-HCl, pH 7.4, 10 mM NaCl, 3 mM $MgCl_2$, 0.1% IGEPAL CA-630) and spun down immediately. Nuclei were

resuspended in the nuclei buffer provided by 10X Genomics, counted and subjected to Tn5 tagmentation. The subsequent steps were done according to the manufacturer's instructions for the 10X Genomics Single Cell ATAC v1.0 or v1.1 Kit. Generated scATAC-seq libraries were paired-end sequenced on a NovaSeq 6000 S2.
For immunohistochemistry, representative tissue blocks containing MUM1-positive myeloma cells were selected. The blocks were sectioned with a standard microtome at 2µm thickness. Subsequently, the slides were dried overnight at room temperature. Immunohistochemical staining for MUM1, CD68, CD4, CD8, CD138, Ki67, CXCL12, CXCL7 and CXCR4 was performed using the automated immunostainer Ventana Benchmark Ultra (Roche, USA). For image acquisition of stained slides the Aperio AT2 slide scanner at 40x magnification and the manufacturer's acquisition software suite were used (Leica Biosystems, Nussloch, Germany).
Flow cytometry: Cryopreserved samples were thawed at 37°C and washed twice in ice-cold 1x PBS. The following antibodies were used for the analysis: CD3, CD4, CD8, CD11b, CD33, CD34, CD45RA, CD38, TCRab, CD90, CD138 and CD271. Measurements were performed on a FACSymphony (BD Biosciences). Dead cells were excluded using eFluor-506 (ThermoFisherScientific, USA).
Enzyme-linked Immunosorbent Assay: For ELISA, either paired dry pellets or viably frozen cells were used. Viably frozen CD138+ BMMCs were thawed and washed twice with 1x PBS. Cells were lysed using ice-cold lysis buffer ABIN0-007-3 (antibodies-online GmbH, Aachen, Germany). After 30 min on ice, the tubes were subjected to ultrasonication for 3x 15s. Protein concentrations were determined using the Bicinchoninic acid reaction (Pierce™ BCA Protein Assay Kit, ThermoFisherScientific, Waltham, Massachusetts, USA) according to the manufacturer's instructions. CXCL7 and CXCL12 were quantified using the Pro-Platelet Basic Protein (Chemokine (C-X-C Motif) Ligand 7) (PPBP) and Chemokine (C-X-C Motif) Ligand 12 (CXCL12) ELISA kits offered by antibodies-online, respectively. Due to limited material, we used between 750-1500 ng total protein per reaction, with matched protein loads for paired samples. Quantifications were performed twice in duplicates.
Medical Imaging: Whole-body 18F-Fluorodeoxyglucose (FDG)–positron emission tomography (PET) computer tomography (CT) was performed with a Biograph mCT, S128 (Siemens Co., Erlangen, Germany) 1 h after injection of 18F-FDG. For attenuation correction of PET data and image fusion, a low-dose attenuation CT (120 kV, 30 mA) was used. An image matrix of 400×400 pixels was used for iterative image reconstruction, which was based on the ordered subset expectation maximization (OSEM) algorithm with two iterations and 21 subsets as well as time of flight (TOF). The reconstructed images were converted to SUV images based on the formula: SUV=tissue concentration (Bq/g)/(injected dose (Bq)/body weight (g)). Magnetic resonance imaging (MRI) was performed with a 1.5 Tesla MRI Scanner (Siemens Co., Erlangen, Germany). Imaging sequences comprised coronal T1-weighted turbo spin echo (T1w; repetition time 528 ms, echo time 8.4 ms, in plane resolution 1.3 mm x 1.3 mm, slice thickness 5.0 mm, 10% distance factor), coronal T2 weighted short-TI inversion recovery (T2w; repetition time 3650, echo time 56 ms, in plane resolution 0.7 mm x 0.7 mm (interpolated), slice thickness 5.0 mm, 10% distance factor, fat suppression: slice-selective inversion recovery (TI=160ms)), and axial diffusion weighted imaging (DWI; Diffusion-EPI iShim, repetition time 5130, echo time 64 ms, in plane resolution 1.8 mm x 1.8 mm (interpolated), slice thickness 6.0 mm, 0% distance factor, b values: 50 s mm-2 and 800 s mm-2, fat suppression: slice-selective inversion recovery (TI=180ms)). For PET, a focal lesion was defined as a circumscribed focus with increased FDG uptake compared with its surroundings. For MRI, focal lesions were defined as focal hypointensity in T1 and as hyperintensity in T2 and the high b-value image from DWI. The median diameter of the analysed focal lesions was 2.6 cm (range: 1.4-7.8 cm). Guided sampling was performed using a Siemens Emotion 16 CT (Siemens Co., Erlangen, Germany) or by surgical resections. The number and size of osteolytic lesions is given in Suppl. Data 8.

Data analysis

WGS: Raw sequencing data was processed and aligned to human reference genome build 37 version hs37d5 (1000 Genomes Project) using the DKFZ OTP WGS pipeline. Copy number aberrations (CNAs) were identified using ACEseq (v1.2.8-4), and single nucleotide variants (SNVs) using samtools mpileup (v1.2.166-3). For the SNVs additional filtering steps were applied, including blacklist filtering, fpfilter (https://github.com/genome/fpfilter-tool, v1.0) and removal of SNVs located in regions coding for immunoglobulins. For SNVs, which were only called in one of the paired samples, Rsamtools (v2.6.0) was used to determine the number of reference and variant reads in both samples. In addition, we performed manual somatic variant refinement using IGV (v2.7.2) according to a published standard operating procedure and cancer clonal fractions (CCF) were calculated.
GEP: As chip definition file (CDF) we used the Affymetrix U133 Version 2.0 plus array custom (CDF) mapping to Entrez genes (http://brainarray.mhri.med.umich.edu/Brainarray/Database/CustomCDF/). Expression data were normalized using GC-RMA and converted to log2 scale.
RNA-seq: Paired-end reads were mapped to the STAR index generated reference genome (build 37, hs37d5) using STAR v2.5.2b. Gene expressions were quantified using featureCounts (Subread v1.5.1). Differential gene expression analysis was performed with the R-package DESeq2 (v1.28.1) and regularized logarithm (rlog) function was used for transformation to log2 scale and normalization including library size. Differentially expressed genes were visualized using the R-package EnhancedVolcano (https://github.com/kevinblighe/EnhancedVolcano, v1.6.0).
scRNA-seq: For each demultiplexed library CellRanger count (v5.0.0) was run with reference refdata-gex-GRCh38-2020-A to quantify single cell feature counts. Cellranger vdj (v5.0.0) was used for each single TCR and BCR library (with reference refdata-cellranger-vdj-GRCh38-alts-ensembl-5.0.0). The count matrices were loaded into R (v4.0.2) by using the standard Seurat (v4.0.1) parameters and annotated for patient, location (focal lesion/RBM), sorting fraction and then merged all together. Cells with more than 5% mitochondrial RNA, less than 200 or more than 5000 expressed genes were removed (Suppl. Fig. 3a, Suppl. Table 7). Cell doublets were identified and removed using Scrublet (v0.2.3, prediction score > 0.3). Immunoglobulin genes were removed from the dataset. Normalization was done for each sample using SCtransform and technical or biological confounding effects such as mitochondrial counts or cell cycle stages were regressed out using the "vars.to.regress" argument. Harmony (v1.0) was used for integration of multiple datasets. All samples were clustered together based on Seurat k-nearest neighbors clustering with a resolution of 0.5 and cells were embedded into a two-dimensional space using Uniform Manifold Approximation and Projection (UMAP) (Suppl. Fig. 3b). For further analysis, the dataset was split into a tumor and a TME dataset (Suppl. Fig. 3c+d). The cell type assignment of the TME was done based on a Cite-seq BM reference dataset by using the multimodal reference mapping approach from Stuart et al. In brief, the cell type assignment of each sample works as follows: anchors were defined between the reference and each query sample and then each sample was individually mapped to the reference. In a next step, all annotated samples were merged as they have been integrated into a common reference space and then visualized. The tumor cells of the CD138-negative fraction, respectively the TME cells from the CD138-positive fraction were removed from downstream analysis. The separate tumor and TME datasets were then again normalized with SCtransform. Cell-cycle scores were calculated with the Seurat function "CellCycleScoring". Differential expression analysis was performed with the standard Seurat function Find(All)Markers (parameters: min.pct = 0.25, logfc.threshold = 0 for subclone comparison between focal lesions and RBM, respectively logfc.threshold = 0.25 for the general subclone comparisons). Pearson correlations between subclones were calculated based on the genes, which were also used for the differential expression analysis (min.pct = 0.25, logfc.threshold = 0). For gene set enrichment analysis the hypeR R-package (v1.6.0) was used with the MSigDB HALLMARK geneset. Gene expression signatures were calculated with the Seurat function AddModule Score (https://satijalab.org/seurat/index.html) by using recently published signatures to determine the level of exhaustion (TIGIT, HAVCR2, CTLA4, PDCD1, LAG3, LAYN) and cytotoxicity (NKG7, CCL4, CST7, PRF1, GZMA, GZMB, IFNG, CCL3) of T-cells.
VDJ filtered_contig_annotations files were loaded into R, combined from all patients (separately for TCR and BCR) and mapped to the Seurat object by using the scRepertoire package (v0.99.15). Clonal T-cells, which made up ≥1% of the total T-cell population and showed at least 5 cells in one of the paired samples, were called expanded. Clonal T-cells making up ≥5% of all T-cells were called hyperexpanded. T-cells with

incomplete receptor sequences (only one successfully sequenced chain) were merged with the corresponding clonal T-cells with complete chain information. To test for antigen specificity, the CDR3 sequences of the expanded TCRs were looked up in the curated databases VDJdb and McPas-TCR.
To call mutations in scRNA-seq data, SNVs positions from the WGS data (hg19) were transformed to hg38 using rtracklayer (v1.50.0) and mutation calling in the scRNA-seq data was performed using Vartrix with default parameters (https://github.com/10XGenomics/vartrix). This mutation information was then mapped to the Seurat tumor objects.
scATAC-seq: Raw scATAC-seq reads were aligned to the reference genome GRCh38 using CellRanger ATAC (10X Genomics, version 1.2). For downstream analysis, the fragment files of all samples (4x paired focal lesion and random bone marrow specimens) were loaded into the ArchR framework (v1.0.2) with default parameters including doublet detection. In addition, based on the quality control plots all cells with a transcription start site (TSS) enrichment score < 8, < 3000 fragments, a doublet enrichment score >6 and a predicted doublet score >200 were filtered out (Suppl. Fig. 3e-g, Suppl. Table 8). This resulted in 7741 cells with a median TSS of 13.726 and median fragment size of 18,239. First, all cells from the different patients together were normalized using iterative latent semantic indexing (LSI) and clustered with a resolution of 0.5 (Suppl. Fig. 3h). Gene activity imputation was performed using MAGIC and peak calling with MACS2 (v2.2.6, extendSummits = 750).
Plasma/myeloma cells could be distinguished from other immune cells based on the gene activity score of CD138/SDC1. One cluster containing plasma cells from different patients was defined as a normal plasma-cell reference for CNA-calling analogous to scRNA-seq (Suppl. Fig. 3h). Motif deviations were calculated based on the JASPAR database. For pseudobulk TF motif analysis, the MotifMatrix was extracted from the ArchR-project using the "getMatrixFromProject" function. For each TF motif, a mean value was calculated over all cells from the corresponding sample. The most highly variable TFs were visualized using pheatmap (v1.0.12) in R. Co-accessibility between genomic regions was separately calculated for timepoints and CNA subclones, respectively, adjusting the ArchR framework to single-cell resolution without aggregation of cells. The degree of co-accessibility in the background was determined by randomly shuffling the accessibility values over cells and peaks as described previously. The 99th percentile of the maximum shuffled background co-accessibility score was used as a threshold to determine true co-accessible links. Co-accessible links were further evaluated by percent of accessible cells in the linked peak pairs.
Tumor subclone calling: For scRNA-seq, subclones were identified based on the presence of subclonal CNAs. CNAs were predicted using the R-package InferCNV (v1.6.0). The prediction is based on expression averages of adjacent genes over large genomic regions. As reference we used normal plasma cells from patients in our dataset, which clustered together (Suppl. Fig. 3d). As input for InferCNV we used normalized counts of the patients together with a gene ordering file built based on the 10X Genomics reference file for running CellRanger. Importantly, we only included regions with subclonal CNAs according to WGS. The cells were hierarchically clustered (method ward.D2) based on the InferCNV output and the clustered dendrogram was cut according to visually identified subclones using the R-package dendextend (v1.15.2).
For scATAC-seq, CNAs were called using a script published by Lareau et al. (https://github.com/caleblareau/mtscATACpaper_reproducibility/tree/master/cnv_compute). Briefly, overlapping 10 Mb genome-wide bins were constructed from the fragments file. For all cells passing QC criteria a bin by cell matrix was computed separately for the malignant and the normal plasma cells from all patients, and a z-score matrix was calculated using the normal plasma cells as reference. Next, similar to the scRNA-seq approach, we performed hierarchical clustering of regions with subclonal CNAs according to WGS. Heatmaps of clustered z-scores for chromosomes from single cells were visually inspected and compared to scRNA-seq heatmaps and WGS chromosomal profiles to identify congruities and conflicting results. The location of chromosomal regions, which were used for clustering, and the number of consecutive rounds, in case an iterative process was required, are shown in Suppl. Table 9. The recently published script for the WGS-guided subclone identification in scRNA-seq and scATAC-seq data is available at: https://github.com/a-poos/MM_subclones.
Publicly available IRF4 ChIP-seq data for MM cell line KMS12BM were downloaded from the European Nucleotide Archive (accession number: PRJEB25605; https://www.ncbi.nlm.nih.gov/bioproject/PRJEB25605/) and peaks were called using the nfcore/chipseq pipeline (v.1.0.0) with default settings (narrowPeaks).
Immune histochemistry: Images were analyzed using the QuPath software (v0.3.2). Therefore, the images were imported using the Bioformats builder. Detection of all cells and the positive stained cells within the specimen was done using the integrated "positive cell detection" module of QuPath with "Hematoxylin" as the reference channel.
Flow cytometry data was analysed using BD FACSDiva (version 9.1) and FlowJo (version 10.9.0).
Statistical methods: Statistical analyses were carried out using the R software package v4.0.2. Group comparisons of continuous variables were done using the two-sided Wilcoxon rank sum test for unpaired samples and the two-sided Wilcoxon signed rank test for paired samples. The Kaplan-Meier method was used for survival analyses. Overall survival was time from enrollment to death of any cause. Cox proportional hazards regression was used to estimate hazard ratios and 95% confidence intervals.

For manuscripts utilizing custom algorithms or software that are central to the research but not yet described in published literature, software must be made available to editors and reviewers. We strongly encourage code deposition in a community repository (e.g. GitHub). See the Nature Portfolio guidelines for submitting code & software for further information.

Data

Policy information about availability of data

All manuscripts must include a data availability statement. This statement should provide the following information, where applicable:

- Accession codes, unique identifiers, or web links for publicly available datasets
- A description of any restrictions on data availability
- For clinical datasets or third party data, please ensure that the statement adheres to our policy

WGS, RNA, scRNA, scATAC, TCR and BCR sequencing data of this study have been deposited at the European Genome-phenome Archive with the study identifier EGAS00001006090 (https://ega-archive.org/studies/EGAS00001006090) and are available on request from the associated Data Access Committee (hipo_daco@dkfz-heidelberg.de) due to them containing patient information under controlled access. Access will be granted to commercial and non-commercial parties according to patient consent forms and data transfer agreements. We have an institutional process in place to deal with requests for data transfer and aim for a rapid response time. The duration of data access after approval is limited to 36 months.
The publicly available gene expression profiling datasets used in this study are available at ArrayExpress under accession code E-MTAB-2299 (https://www.ebi.ac.uk/biostudies/arrayexpress/studies/E-MTAB-2299) and Gene Expression Omnibus under accession code GSE19784 (https://www.ncbi.nlm.nih.gov/geo/query/acc.cgi?acc=GSE19784). The publicly available whole exome sequencing data used in this study are available from the European Genome-phenome Archive under accession code EGAS00001002111 (https://ega-archive.org/studies/EGAS00001002111). IRF4 ChIP-seq data of the MM cell line KMS12BM used in this study are publicly available from the European Nucleotide Archive with the accession number PRJEB25605 (https://www.ncbi.nlm.nih.gov/bioproject/PRJEB25605/). The remaining data are available within the Article, Supplementary Information or Source Data file. Source data are provided with this paper.

Research involving human participants, their data, or biological material

Policy information about studies with human participants or human data. See also policy information about sex, gender (identity/presentation), and sexual orientation and race, ethnicity and racism.

Reporting on sex and gender	From the 31 patients in our set of paired samples 4 and 27 had female and male sex, respectively. Sex was determined based on self-report and in all subjects corresponded to assigned sex. Sex was not considered in the initial study design due to the small number of eligible patients.
Reporting on race, ethnicity, or other socially relevant groupings	All 31 patients included in this study were Caucasians.
Population characteristics	This study included newly diagnosed treatment-requiring Multiple Myeloma patients presenting at the Heidelberg University clinic. Patients' characteristics, the origin of samples and the analyses, which were performed with the respective samples, are shown in Fig. 1b and Suppl. Tables 3-4. Study subjects did not receive compensation for participation in the study. Due to the intra-patient comparison and the small number of available subjects sex or gender was not considered in the study design. Sex was determined by self-report and in all cases corresponded with assigned sex at birth.
Recruitment	We included patients with accessible focal lesions, for whom sufficient available material for sequencing or immune histochemistry stainings of tumor and/or microenvironment cells from the iliac crest and a focal lesion sample were available. There is an enrichment of male gender in our set of patients with paired samples (27/31 patients). While myeloma in general is more often seen in male patients (~60% male), we cannot exclude that male patients were more likely to participate in our study, which involved additional imaging and invasive procedures. However, to the best of our knowledge, there is no evidence for fundamental differences in myeloma biology between males and females. Furthermore, in the UAMS and the GMMG data set, which we used to confirm expression differences between paired samples and to address the clinical implications of differential gene expression, the proportion of male patients was ~60%. Therefore, we are confident that our findings are representative for spatial heterogeneity in this disease.
Ethics oversight	Heidelberg University Medical Faculty Ethics Board (S278-13)

Note that full information on the approval of the study protocol must also be provided in the manuscript.

Field-specific reporting

Please select the one below that is the best fit for your research. If you are not sure, read the appropriate sections before making your selection.

☒ Life sciences ☐ Behavioural & social sciences ☐ Ecological, evolutionary & environmental sciences

For a reference copy of the document with all sections, see nature.com/documents/nr-reporting-summary-flat.pdf

Life sciences study design

All studies must disclose on these points even when the disclosure is negative.

Sample size	As an exploratory study, no sample size calculation was performed. Our set included 17 patients with newly diagnosed Multiple Myeloma (plus 14 additional patients for validation experiments with flow cytometry and immune histochemistry stainings).
Data exclusions	No paired WGS, bulk RNA and scRNA-seq data was available for P06, because CD138 positive cells could not be evaluated in the random bone marrow sample. The sample for WGS and bulk RNA-sequencing was lost due to technical difficulties and the single-cell sample was lost during library preparation due to a partial clog. Therefore, for this patient only microenvironment data was available. No RNA for bulk RNAseq could be extracted for P08, P09, P10, P14 and P15 due to technical reasons. Patients P18-P31 were only used for validation purposes. For patients P18-P20 only flow cytometry data was available, while for patients P21-P31 only stamps for immune histochemistry staining were available.
Replication	Since myeloma is a rare disease and the study is based on a unique dataset, there is no replication.
Randomization	There was no randomization in this exploratory study. Since we focused on intra-patient comparisons and did not compare treatments, specific subgroups or other interventions, randomizing patients was not relevant to our study.
Blinding	The expert pathologist A.B. was blinded to the results of single cell sequencing when analyzing the immunohistochemistry data. During collection and quality control of sequencing data the respective investigator was blinded to group allocation. Furthermore, the investigator, who quantified proteins using ELISAs, was blinded to sample type, too. For the analysis of spatial heterogeneity between focal lesions and random bone marrow no blinding was possible, since this comparison was the major aim of our study.

Reporting for specific materials, systems and methods

We require information from authors about some types of materials, experimental systems and methods used in many studies. Here, indicate whether each material, system or method listed is relevant to your study. If you are not sure if a list item applies to your research, read the appropriate section before selecting a response.

Materials & experimental systems

n/a	Involved in the study
☐	☒ Antibodies
☒	☐ Eukaryotic cell lines
☒	☐ Palaeontology and archaeology
☒	☐ Animals and other organisms
☒	☐ Clinical data
☒	☐ Dual use research of concern
☒	☐ Plants

Methods

n/a	Involved in the study
☒	☐ ChIP-seq
☐	☒ Flow cytometry
☒	☐ MRI-based neuroimaging

Antibodies

Antibodies used

Immunohistochemical staining: MUM1 (mouse anti-human clone MUM1P, Agilent DAKO, Santa Clara, USA, v M7259, dilution 1:50), CD68 (mouse anti-human clone KP-1, Roche Ventana, USA, Cat# 790-2931, ready-to-use), CD4 (rabbit anti-human clone SP35, Roche Ventana, USA, Cat# 790-4423, ready-to-use), CD8 (rabbit anti-Human, clone SP57 Roche Ventana, USA, Cat# 790-4460, ready-to-use), CD138 (mouse anti-human clone B-A38, Roche Ventana, USA, Cat# 760-4248, ready-to-use), Ki67 (clone 30-9 , rabbit anti-human, Roche Ventana, USA, Cat# 790-4286, ready-to-use), CXCL12 (rabbit anti-human, polyclonal, LSBio, Seattle, USA, Cat# LS-B7489, dilution 1:40), CXCL7 (rabbit anti-human, polyclonal, LSBio, Seattle USA, Cat# LS-B7394, dilution 1:20) and CXCR4 (mouse anti-human, clone 12G5, LSBio, Seattle, USA, Cat# LS-B1986, dilution 1:50).
Flow cytometry: CD3 (mouse anti-human clone SK7, Cat# 740832, dilution 1:100 and Cat# 560176, dilution 1:20), CD4 (mouse anti-human clone SK3, Cat# 563550, dilution 1:100), CD8 (mouse anti-human clone SK1, Cat# 741199, dilution 1:100), CD11b (rat anti-human clone M1/70, Cat# 751140, dilution 1:50), CD33 (mouse-anti human clone WM53, Cat# 551378, dilution 1:20), CD34 (mouse anti-human clone 581, Cat# 555822, dilution 1:5), CD38 (mouse anti-human clone HIT2, Cat# 551400, dilution 1:100), CD45RA (mouse anti-human clone HI100, Cat# 564552, dilution 1:100), CD90 (mouse anti-human clone 5E10, Cat# 747750, dilution 1:50), CD138 (mouse anti-human clone MI15, Cat# 749874, dilution 1:50), TCRab (mouse anti-human clone IP26, Cat# 747180, dilution 1:50) (all BD Biosciences, Franklin Lakes, USA) and CD271 (mouse anti-human, clone ME20.4-1.H4, Miltenyi-Biotech, Germany, Cat# 345110, dilution 1:50).
Please see Suppl. Table 6 for more detailed information about the used antibodies.

Validation

Immune histochemistry: The antibodies CD4, CD8, CD68, MUM1, CD138 and Ki67 are all CE and IVD certified for human. In Heidelberg they are used in pathological routine diagnostics with the appropriate controls for validation and verification of staining. CXCL12, CXCL7 and CXCR4 are commercially available, broadly established and validated by the manufacturers for immunohistochemistry application on websites and technical data sheets. As controls, Dr. Brobeil used specimens from tonsil, liver and pancreas.
Flow cytometry: All antibodies are commercially available, broadly established and validated by the respective manufacturers as indicated on the websites. In addition, used antibodies are used routinely in our laboratory with reproducible results.
Please see also Suppl. Table 6 for more details.

Flow Cytometry

Plots

Confirm that:

☒ The axis labels state the marker and fluorochrome used (e.g. CD4-FITC).

☒ The axis scales are clearly visible. Include numbers along axes only for bottom left plot of group (a 'group' is an analysis of identical markers).

☒ All plots are contour plots with outliers or pseudocolor plots.

☒ A numerical value for number of cells or percentage (with statistics) is provided.

Methodology

Sample preparation

Cryopreserved samples were thawed at 37°C and washed twice in ice-cold 1x PBS.

Instrument

Measurements were performed on a FACSymphony (BD Biosciences) and cell-sorting was done using BD Aria II.

Software

BD FACSDiva and FlowJo

Cell population abundance

Purity in post sort fractions was not directly determined. Post-sort WGS was performed, which gave detailed insights into tumor purity.

Gating strategy

Dead cells were excluded using eFluor-506 (ThermoFisherScientific, USA). Plasma cells were identified as CD38+ CD138+ and were excluded from further gating. Monocytes/macrophages were defined as CD11b+ CD33+, mesenchymal stromal cells as CD90+ CD271+ and CD8+ T-cells as CD3+, TCRab+ and CD8+. Gating strategy is provided in Suppl. Fig. 9.

☒ Tick this box to confirm that a figure exemplifying the gating strategy is provided in the Supplementary Information.
